# Partial Enteral Nutrition Mitigated Ischemia/Reperfusion-Induced Damage of Rat Small Intestinal Barrier

**DOI:** 10.3390/nu8080502

**Published:** 2016-08-17

**Authors:** Chao Wu, Xinying Wang, Tingting Jiang, Chaojun Li, Li Zhang, Xuejin Gao, Feng Tian, Ning Li, Jieshou Li

**Affiliations:** 1 Research Institute of General Surgery, Jinling Hospital, Medical School of Nanjing University, Nanjing 210002, China; wuchao0008@126.com (C.W.); chinesemilk@126.com (T.J.); zlshe1107@163.com (L.Z.); cyyywcwk@163.com (X.G.); tianfeng_nju@163.com (F.T.); ptwklining@163.com (N.L.); 15005171483@sina.cn (J.L.); 2 Department of Gastrointestinal Surgery, The First Affiliated Hospital of Chongqing Medical University, Chongqing 400016, China; 3 Jiangsu Key Laboratory of Molecular Medicine, Medical School of Nanjing University and Model Animal Research Center, National Resource Center for Mutant Mice, Nanjing 210093, China; ptwkwuchao@163.com

**Keywords:** partial enteral nutrition, ischemia-reperfusion injury, hypoxia-inducible factor, intestinal epithelial barrier function

## Abstract

*Background and Aims*: This study was designed to investigate a relatively optimum dose of partial enteral nutrition (PEN) which effectively attenuates intestinal barrier dysfunction initiated by ischemia/reperfusion injury (IRI). *Methods*: In experiment 1, 60 male Sprague-Dawley (SD) rats were subjected to intestinal IRI and assigned to six groups according to the different proportion of EN administrations: namely total parenteral nutrition (TPN or 0%EN), 10%EN, 20%EN, 40%EN, 60%EN, and total enteral nutrition (TEN or 100%) groups, the deficits of intraluminal calorie were supplemented by PN. In experiment 2, 50 male SD rats were subjected to intestinal IRI and divided into five groups based on the results of experiment 1: TPN, TEN, 20%EN, TPN plus pretreatment with NF-κB antagonist 30 min before IRI (TPN+PDTC), and TPN plus pretreatment with HIF-1α antagonist 30 min before IRI (TPN+YC-1) groups. *Results*: In experiment 1, previous IRI combined with subsequent EN shortage disrupted the structure of intestinal epithelial cell and tight junctions (TJs). While 20% dose of EN had an obviously protective effect on these detrimental consequences. In experiment 2, compared with TPN only, 20%EN exerted a significant protection of barrier function of intestinal epithelium. Analogous results were observed when TPN combined with specific NF-κB/HIF-1α inhibitors (PDTC and YC-1). Meanwhile, the expression of NF-κB/HIF-1α had a similar trend among the groups. *Conclusions*: Our findings indicate that 20%EN is the minimally effective dosage of EN which promotes the recovery of intestinal barrier function after IRI in a rat model. Furthermore, we discreetly speculate that this benefit is, at least partly, related to NF-κB/HIF-1α pathway expression.

## 1. Introduction

Ischemia reperfusion injury (IRI) is a pathophysiologic process in which hypoxic organ damage is deteriorated following restoration of blood flow and oxygen delivery to the ischemic tissue [1,2,3]. The intestinal mucosal epithelium is susceptible to decreased mesenteric blood flow after the interruption of oxygen and nutrients [4]. If the ischemic factors are not promptly removed, reperfusion may cause additional damage to the intestine. Epithelial cell death and shedding causes dysfunction of intestinal barriers and toxic gut-derived substances enter the mesenteric lymph and blood flow to induce remote organ injury [5]. Therefore, the integrity of the gastrointestinal barrier is closely associated with homeostasis in the host. Intestinal IRI is considered to be a “motor” of systemic inflammatory response syndrome and multiple organ dysfunction syndrome [6,7].

Sensing and coordinating responses to hypoxia during ischemia are primarily facilitated by the transcription factor hypoxia-inducible factor (HIF) [8]. HIF-1 is an essential determinant in the pathophysiological response to hypoxia/ischemia [8]. HIF-1 is known as a heterodimer of an O_2_-regulated α-subunit and a constitutively expressed β-subunit, which are both members of the bHLH/PAS transcription factor family [8,9]. Physiologically, the abundance of α-subunits is primarily regulated by a family of prolyl hydroxylases (PHDs) consisting of PHD1, PHD2, and PHD3. Under normoxic conditions, PHDs catalyze the hydroxylation of specific prolyl residues within the oxygen-dependent degradation domain of HIF-1α. Next, the von Hippel–Lindau tumor suppressor protein binds to HIF-1α for subsequent ubiquitin-proteasomal degradation. Under hypoxia conditions, PHDs activity is decreased, causing HIF-1α to become stabilized and accumulate in the cytoplasm, enter the nucleus, and dimerize with HIF-1β [9,10]. Consequently, HIF-1 dimer protein levels are remarkably upregulated. Previous studies showed that proinflammatory cytokines and lipopolysaccharide (LPS) might induce persistent expression of HIF-1α even under normoxic conditions. The nuclear factor (NF)-κB pathway plays a key role in the pathogenesis of LPS- and cytokine-induced HIF-1α activation. Furthermore, NF-κB is one of the most recognized transcription factors regulating HIF-1α in the inflammatory microenvironment such as in inflammatory bowel disease [11]. Harki et al. found that HIF-1α is expressed not only in acute and chronic ischemic tissues, but also in normal colon tissue and inflammatory disorders such as infectious colitis and inflammatory bowel disease [12]. A study of a dextran sulfate sodium-induced colitis model confirmed that knockout of HIF-1α in dendritic cells led to more severe intestinal inflammation, increased levels of proinflammatory cytokines, and enhanced production of mucin [13]. These results indicated that HIF-1α may have a potentially protective effect in inflammation of intestinal tissue. However, apparent discrepancies in previous literature still exist. In cell culture experiments in vitro, Yang et al. [14] found that interferon-γ induces epithelial barrier dysfunction and the disruption of tight junctions (TJs) by upregulating HIF-1α expression through the NF-κB pathway. Lei et al. [15] reported that inhibiting the NF-κB and HIF-1α signaling pathways attenuated LPS- and hypoxia/reoxygenation-induced intestinal epithelial barrier dysfunction. In an animal study using wild-type and HIF-1α^+/−^ mice, Kannan et al. [16] showed that HIF-1α activation is involved in superior mesenteric artery occlusion-induced intestinal barrier dysfunction and mucosal inflammatory response, while partial HIF-1α deficiency attenuates gut IR-induced villus injury.

IRI of the small intestine is a common event in abdominal surgery, trauma, and major burns after fluid resuscitation. For patients undergoing intestinal IRI, total parenteral nutrition (TPN) is preferred to sufficient oral or enteral nutrition (EN) which are difficult to implement. However, insufficient enteral nourishing may lead to reduced growth and functional development of the intestinal epithelium, potentially resulting in the hazardous intraluminal content translocating to the blood and lymph fluids [17,18,19]. The protective effect of enteral feeding in the gut has been investigated in human and animal models of hemorrhage or viscera IRI in numerous studies [20,21,22]. Particularly, under IRI conditions, deficiency of EN may aggravate intestinal epithelial barrier dysfunction [22]. It has been suggested that the beneficial effects of minimal enteral feeding result from the intestinal trophic effect of enteral nutrients [23]. Indeed, it was confirmed that luminal nutrients stimulate intestinal growth and maintain mucosal integrity in adult and neonatal animals [23]. Grossie et al. [24] demonstrated that EN prevented delayed transit induced by non-lethal ischemia in a rat model. Clinically, a number of patients tolerate a partial EN because of imperfect digestive/absorptive function of the gastrointestinal tract. A previous large-sample randomized controlled study showed that a 6-day strategy of initial trophic EN compared with full EN (400 kcal/day vs. 1300 kcal/day) was associated with lower gastrointestinal intolerance for critically ill patients [25]. It is widely known that EN should be used for patients as soon as it can be safely tolerated. However, the minimally effective proportion of EN for sustaining intestinal barrier function remains controversial.

In this study, we performed partial EN (PEN) combined with supplementary PN (SPN) in a rat model of preconditioned intestinal IRI. First, because PN without enteral feeding is associated with a greater risk of infection and impaired gut-derived immune responses, we hypothesized that the deficiency of enteral feeding activates the NF-κB/HIF-1α signaling pathway despite complete reperfusion, leading to impairment of the intestinal mucosa barrier. Second, because TEN may not be tolerated by numerous patients, we investigated the optimized dose of PEN for maintaining intestinal mucosa integrity. Furthermore, we hypothesized that PEN inhibits expression of the NF-κB/HIF-1α signaling pathway in a rat model of intestinal IRI.

## 2. Materials and Methods

### 2.1. Animal Care and Experimental Procedures

This study was approved by the Animal Care and Use Committee of Jinling Hospital, Nanjing, China (No. 2014013). Male Sprague-Dawley rats (6 weeks old), weighing 200–250 g, were used. Rats were acclimated for 1 week to a temperature-controlled room on a 12-h light/dark cycle and the ad libitum chow and water. They were fasted overnight but allowed free access to water until 4 h before laparotomy.

Operative procedures were performed using standard sterile technique under general anesthesia with ketamine (100 mg/kg, i.p.). Rats underwent placement of heparinized silicon rubber catheter (0.012 inch i.d./0.025 inch o.d.; Helix Medical, Inc., Carpinteria, CA, USA) in the vena cava through the right external jugular vein for PN. The catheter was tunneled subcutaneously and exited at the midpoint of the tail [26]. Then a 3-cm incision was made in the skin just below the sternum, and a silicon rubber tube (medical grade, 0.020 i.d.; 0.029 o.d., SF Medical, Hudson, MN, USA) for EN was routed subcutaneously from the midline of the scapular area to the incision. A 3-cm incision was then made through the abdominal muscle and the duodenum exteriorized. A purse-string suture was made in the duodenum, a puncture was carefully made with a thick needle, and 2.5 cm of the tube inserted distally into the duodenum. The tube was fixed to the duodenum around a ridge by medical adhesive (CONPONT Medical, Beijing, China). The duodenum was then carefully replaced into the abdomen [27].

### 2.2. Intestinal IRI Protocol

After the PN and EN access was established, the principal branches of superior mesenteric artery (SMA) were gently separated, occluded by a noninvasive artery clip to conduce to a 30-min ischemic stage. The intestine was then placed back in the abdomen, and the incision was temporarily closed with a hemostat. The rats were placed under a heat pad during the ischemia. After 30 min, the clip was removed and the abdominal incision was closed. Rats were separately housed in metal cages with wire grid floors to eliminate coprophagia, and partially immobilized by tail restraint to protect the catheter during infusion [26,28]. This technique of infusion in the rat has been adopted in several studies to keep the animals from hyperactivity, which may break the tubes.

### 2.3. Experiment 1 Different Dose of EN on Barrier Function NF-κB/HIF-1α Activation after IRI

Sixty IRI rats were randomly divided into 6 groups: TEN, 10%EN + 90%PN, 20%EN + 80%PN, 40%EN + 60%PN, 60%EN + 40%PN, and TPN groups.

All rats were resuscitated by 0.9% saline at a rate of 4 mL/h for 12 h after IRI and free access to water. TEN rats (*n* = 10) received EN solutions (Ensure Nutrison^®^ powder diluted with water as a rate of 25 g/100 mL) via EN tube (2.0 mL/h) and 0.9% saline via PN tube (0.8 mL/h). Ensure Nutrison^®^ (Abbott, Chicago, IL, USA) contains 14.1% protein, 54.2% carbohydrates, 31.7% lipids, electrolytes, and multivitamins, with a non-protein calorie/nitrogen ratio of 153:1 (640 kJ/g nitrogen). TPN rats (*n* = 30) initially received 1.0 mL/h of PN and advanced to 2.2 mL/h by the second day. The PN solution contains 14.3% amino acids, 53.8% dextrose, 31.9% lipids, electrolytes, and multivitamins, with a nonprotein calorie/nitrogen ratio of 635 kJ/g nitrogen. The EN and PN solutions were almost isocaloric and isonitrogenous to ensure the rats had the same nutritional intake. This feeding meets the calculated nutrient requirements of rats weighing 200 to 250 g.

After 6 days of nutrition support, animals were anesthetized and exsanguinated by portal vein puncture. The small intestine and mesenteric lymph nodes were removed, and the mesenteric fat and external vasculature were dissected away. The samples were fixed in paraformaldehyde or frozen in liquid nitrogen and stored at −80 °C for subsequent tests.

### 2.4. Experiment 2 Effect of 20%EN on NF-κB/HIF-1α Inhibition, and Barrier Function Preservation after IRI

The IRI rats were randomly divided into 5 groups: TPN group (*n* = 10), TEN group (*n* = 10), 20%EN group, TPN plus NF-κB antagonist (pyrrolidine dithiocarbamate, PDTC, BioVision) 100 mg/kg given intraperitoneally 30 min before IRI and 50 mg/kg every subsequent day (TPN+PDTC group, *n* = 10), and TPN plus HIF-1α antagonist (3-(50-hydroxymethyl-20-furyl)-1-benzylindazole, YC-1, CAYMAN), 2 μg/kg, given intraperitoneally 30 min before IRI and 10 μg/kg every subsequent day (TPN+YC-1 group, *n* = 10). Both antagonists were diluted in 1% dimethyl sulfoxide PBS in TPN+PDTC and TPN+YC-1 groups. TPN, TEN, and 20%EN groups received the vehicle (dimethyl sulfoxide PBS) intraperitoneally at the same time point.

After 6 days of treatment, epithelial permeability was accessed by in vivo permeability assay under anesthesia. Then the distal small intestine and mesenteric lymph nodes were excised, fixed in paraformaldehyde or frozen in liquid nitrogen and stored at −80 °C.

### 2.5. In Vivo Intestinal Permeability Assay

A midline laparotomy was carried out under anesthesia, and the renal pedicles ligated with noninvasive arterial clip to prevent urinary excretion of circulating 4-kDa fluorescein isothiocyanate-dextran (FD-4; Sigma-Aldrich, St. Louis, MO, USA). Saline (0.8 mL) containing 100 mg/mL FD-4 was injected into the lumen by the EN tube. After 30-min equilibrium, a 1 mL blood sample was collected from the portal vein. Meanwhile, 2 mL normal saline was infused via PN access to replace blood withdrawn to maintain a stable intravascular volume, because hypovolemia may result in untimely mesenteric ischemia. After centrifugation (3000 rpm, 15 min), 0.1 mL of the plasma was diluted 1:10 with Tris-buffered saline, (TBS, pH-10.5) and quantification of plasma FITC-dextran levels was measured by fluorescence spectrometry (HORIBA, FM-4) at an excitation wavelength of 480 nm and emission wavelength of 540 nm.

### 2.6. Lymph Node Endotoxin Analysis

This assay was conducted in sterile conditions according to previous study completed by Zhao et al. [29]. Mesenteric lymph nodes sample was homogenized (0.1 g/mL normal saline) and centrifuged (3000 rpm, 15 min) to collect supernate. We used the Limulus Amebocyte Lysate test kit (Houshiji Cor. Ltd., Xiamen, China) to carry out endotoxin concentration in accordance with the specification. Briefly, the control standard endotoxin was diluted to 0.01 EU/mL, 0.025 EU/mL, 0.05 EU/mL and 0.1 EU/mL in pyrogen free tubes respectively. Afterwards, 100 μL diluted endotoxin was mixed with 100 μL Limulus amoebocyte lysate in the pyrogen free tube and heated at a temperature of 37 °C for 60 min. The mixed sample was mixed again with 100 μL chromogenic substrate and heated at a temperature of 37 °C for another 60 min. Azo reagent No. 1, No. 2, and No. 3 were added to the reaction system to terminate reactions. Five minutes later, the absorbance at 545 nm of the final mixed sample was measured. After the standard curve was performed, absorbance of the tested plasma endotoxin was measured with the same method, and the concentration of plasma endotoxin was calculated from the standard curve.

### 2.7. Total Protein and Nucleoprotein Extracting

Frozen intestinal mucosal samples (0.1 g) were lysed and homogenized in 1 mL of RIPA buffer (50 mM Tris buffer saline, 0.5% deoxysodium cholate, 1 mM EDTA, 150 mM NaCl, 1% NP-40, 1 mM PMSF) for 30 min on ice and centrifugated at 14,000 rpm for 15 min at 4 °C. Supernatants were collected and kept at 80 °C for Western blot analysis.

Total concentration of proteins of tissue were measured by the BCA protein assay kit (Sangon Biotech Co., Shanghai, China), there was an equal protein concentration of 2.5 μg/μL in each sample. The same procedure had been used for extracting nucleoprotein before removing the supernatant. The nuclear part was resuspended in the 4-fold volume of ice-cold nuclear extraction buffer (15 mM Hepes, pH 7.9, 0.4 MNaCl, 1.5 mM MgCl_2_, 5 mM EDTA, 0.5 mM DTT, 0.5 mM PMSF, 0.1% NP-40, 10% glycerol (*v*/*v*)), with protease inhibitors. After incubation for 30 min on ice with intermittent mixing, samples were centrifuged at 13,000× *g* for 15 min. The supernatant was collected for assaying nucleoprotein p65.

### 2.8. Western-Blot (WB)

Proteins were separated by SDS gels and transferred to polyvinylidene difluoride (PVDF) membranes (Bio-Rad, Hercules, CA, USA). The membranes were blocked with 3% bovine serum albumin prepared in TBS (Tris-buffered saline, pH 7.5 containing 0.1% Tween-20) for 1 h and then incubated with antibodies overnight at 4 °C: anti-p65 (1:200. Santa Cruz, CA, USA), anti-IκBα (1:200. Santa Cruz, CA, USA), IKKβ (1:200. Santa Cruz, CA, USA), mucin-2 (Muc-2, 1:500. Abcam, Cambridge, MA, USA), intestinal trefoil factor (ITF, 1:500. Abcam, Cambridge, MA, USA), ZO-1 (1:500. Abcam, Cambridge, MA, USA), Occludin (1:500. Abcam, Cambridge, MA, USA), HIF-1α (1:500. Santa Cruz, CA, USA), HIF-1β (1:500. Santa Cruz, CA, USA), Cleaved-caspase-3 (1:500. Santa Cruz, CA, USA), Claudin-1 (1:500. Abcam, Cambridge, MA, USA), and anti-GAPDH (1:1000. Santa Cruz, CA, USA). The membranes were then washed three times in TBST (50 mM Tris-HCl pH 7.5, 140 mM NaCl, 0.1% Tween) and incubated with secondary antibody at room temperature for 1 h. An enhanced chemiluminescence reagent, ECL Western blotting detection reagent (Tanon, Shanghai, China), was used to make the labeled protein bands detectable with Image System Minichemi (Saizhi, Beijing, China).

### 2.9. Hematoxylin Eosin (HE) Staining

Distal ileum (2 cm) specimens obtained during the rat experiment were immediately fixed in 10% paraformaldehyde and incubated overnight at room temperature. Next, tissue samples were embedded in paraffin and 5-μm sections were cut. Sections were deparaffinized in xylene and rehydrated in graded ethanol to distilled water and stained with hematoxylin and eosin for histological analysis.

### 2.10. Periodic Acid Schiff (PAS) Staining

The tissue sections were cut into 5 μm slices, deparaffinized in xylene and rehydrated in graded ethanol to distilled water. The samples were then stained with periodic acid-Schiff stain (SBJ-Bio, Nanjing, China) according to the manufacturer’s instructions.

### 2.11. Transmission Electron Microscopy (TEM)

The ileal segments were taken from a similar position in every rat and were fixed with 2.5% glutaraldehyde for 2 h, then post-fixed with 1% OsO4, embedded in Pon812. Ultrathin sections were dislodged and stained with uranyl acetate and lead citrate. Then samples were observed by transmission electron microscopy (Tecnai G2 Spirit, Hong Kong, China) at 80 kV at a magnification of 20,000 and images were recorded. Ultrastructure was observed at the site of microvilli and TJ complex.

### 2.12. Immunohistochemistry (IHC) Assay

For immunohistochemistry, sections were deparaffinized in xylene and rehydrated in graded ethanol to distilled water. Subsequently, the sections were processed using the DAB Detection Kit (ZSGB-Bio, Beijing, China) according to the manufacturer’s protocol. The IHC features shown in the figures are representative of all tissue samples studied. The primary antibodies are as following: HIF-1α (Abcam; 1:100 dilution); Cleaved-caspase-3 (Abcam; 1:200 dilution).

### 2.13. Immunofluorescent (IF) Assay

Frozen sections were cut at 10 μm, and mounted on the slides. Antigen was retrieved by incubating the slides in 10 mM sodium citrate (pH 6) at 90 °C–100 °C. The nonspecific background was blocked by incubation with 5% bovine serum albumin plus 5% newborn bovine serum in PBS for 30 min at room temperature. The sections were incubated with rabbit polyclonal antibody against ZO-1 (Abcam, 1:200 dilution), rabbit polyclonal antibodies against occluding (Abcam, 1:200 dilution), rabbit polyclonal antibody against claudin-1 (Abcam, 1:200 dilution) and p65 (Santa Cruz Biotechnology, Dallas, TX, USA) at 4 °C overnight. The sections were probed with FITC-conjugated or PE-conjugated (only for p65) secondary IgG antibodies. The nuclei were counterstained with DAPI (4,6-diamidino-2-phenylindole, Cell Signaling Technology, Beverly, MA, USA). Slides incubated without any primary antibody were used as negative controls. Confocal analysis was performed with a confocal scanning microscope (BX25, Olympus, Tokyo, Japan).

### 2.14. Enzyme-Linked Immunosorbent Assay (ELISA)

We utilized a commercial ELISA kit from R&D Inc. (Minneapolis, MN, USA) to determine the TNF-α, IL-6, IL-4, IL-10 concentrations of intestinal tissue, which reflect the inflammatory levels. The procedure was in accordance with the manufacturer’s instructions.

### 2.15. Statistical Analysis

All data are presented as mean ± SD. SPSS version 17.0 Software and Prism 5.0 for Windows were used to perform the statistical analysis. The non-parametric Manne-Whitney U test was used to compare continuous variables. *p* < 0.05 was considered as significant.

## 3. Results

### 3.1. Experiment 1

#### 3.1.1. The 20%EN (40 kcal/kg/Day) Down-Regulated NF-κB/HIF-1α

**Figure 1:**
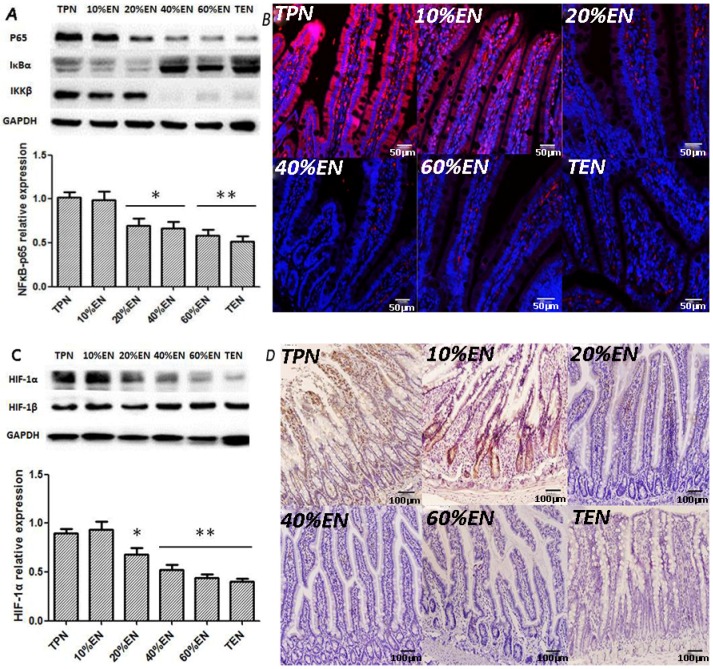
The comparison of NF-κB/HIF-1 expressions in the intestine mucosa between groups. (**A**) Western blotting analysis of p65 protein; (**B**) Immunofluorescence analysis of p65 (red) and DAPI (blue). Compared with TPN group, 20% and 40%EN had a significantly lower expression of p65 protein (*p* = 0.033 and 0.030, respectively). This downregulation was more apparently observed as the EN up to 60% and 100% (*p* = 0.008 and 0.006, respectively); (**C**) Western blotting analysis of HIF-1α and β subunit; (**D**) Immunohistochemical detection of HIF-1α. Likewise, no difference of HIF-1α expression was found between TPN and 10%EN groups. However, 20%EN group had a visibly decreased HIF-1α abundance compared with TPN group (*p* = 0.041). Also, in 40%, 60% and 100%EN groups, an even more significant decrease was found compared with TPN group (*p* = 0.009, 0.007 and 0.007, respectively). No statistical difference of HIF-1β was found between all the groups. * *p* < 0.05, compared with TPN group; ** *p* < 0.01, compared with TPN group.

After 6-day treatment, phosphorylated NF-κB proteins (p65) were significantly increased in the TPN and 10%EN groups compared with other groups (Figure 1A,B). Meanwhile, HIF-1α was significantly high expressed in TPN and 10%EN groups (Figure 1C,D). However, HIF-1β showed no difference between all the groups as expected (Figure 1C). 20%EN and 40%EN rats significantly reduced in expression (*p* < 0.05) and in 60%EN and TEN rats, this difference (*p* < 0.01) was more significant. Inhibitor of NF-κB (IκB) α, as a reverse regulator of p65, had a relative lower expression in TPN, 10%EN, and 20%EN groups. On the contrary, inhibitor of NF-κB kinase (IKK) β as an activator of p65 had an increasing trend in TPN, 10%EN, and 20%EN rats (Figure 1A).

#### 3.1.2. The 20%EN Up-Regulates Intestinal Claudin-1 Protein, Protects Microvilli and Preserves Morphology after IRI

**Figure 2:**
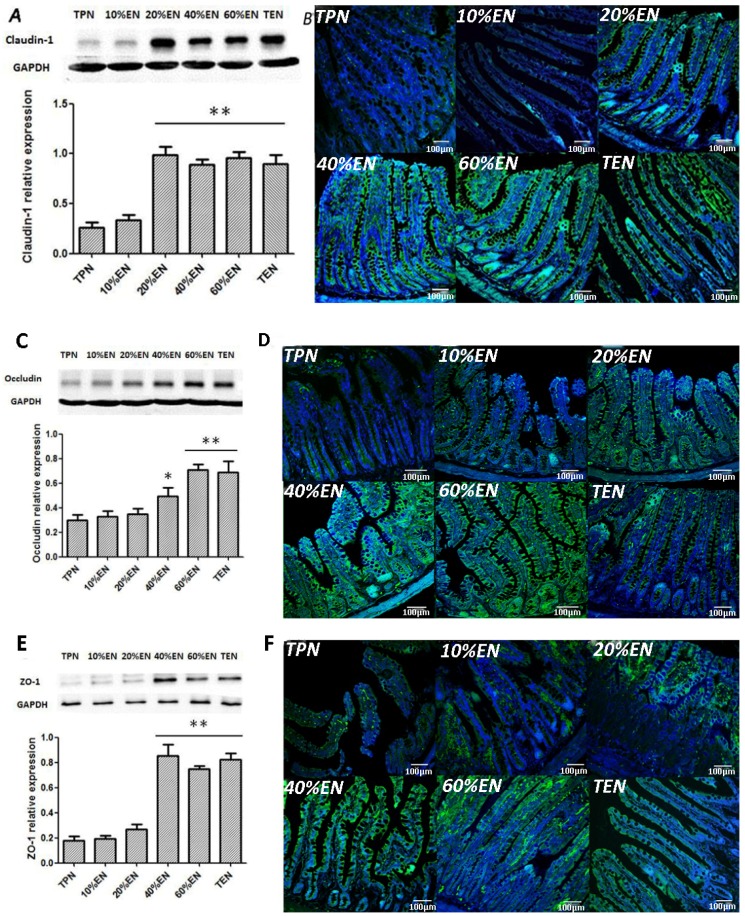
The expression of claudin-1, occludin and ZO-1 protein. (**A**) Western blotting analysis of claudin-1 protein. Apparently, 20% and more EN groups had an increased claudin-1 concentration compared with TPN (*p* = 0.006, 0.006, 0.006, and 0.005, respectively). No statistical difference was found between TPN the 10%EN groups; (**B**) Immunofluorescence analysis of claudin-1 (green) and DAPI (blue); (**C**) Western blotting analysis of occludin. Compared with TPN, it seems that 40% is the minimally effective EN dose for raising the occludin expression (*p* = 0.028). In 60% and 100%EN, more significant increase was found (*p* = 0.006 and 0.007); (**D**) Immunofluorescence analysis of occludin (green) and DAPI (blue); (**E**) Western blotting analysis of ZO-1 protein. Similar trend of ZO-1 expression was found between groups (*p* = 0.007, 0.009, and 0.009 compared with the TPN group). Furthermore, it seems that 40%EN had the same efficiency in maintenance of ZO-1 abundance compared with 60% and TEN; (**F**) Immunofluorescence analysis of ZO-1 (green) and DAPI (blue). * *p* < 0.05, compared with TPN group; ** *p* < 0.01, compared with TPN group.

**Figure 3:**
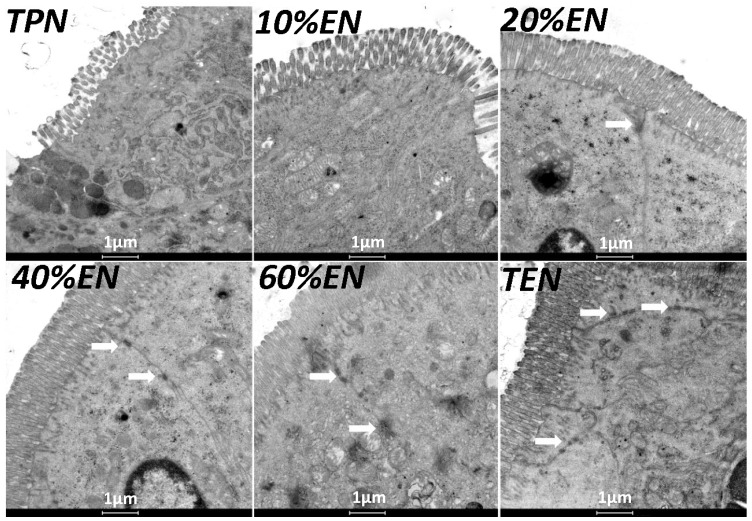
Transmission electron microscopy of intestine of all the groups. This demonstrated that microvilli were visibly damaged and atrophic in TPN and 10%EN groups. While in 20 and more EN dose groups, microvilli had relatively normal integrity. TJ (white arrow) were also relatively intact in 20%, 40%, 60%, and TEN groups. In TPN and 10%EN groups, TJ protein complex was hardly observed compared with the 20% and more EN dose groups.

**Figure 4:**
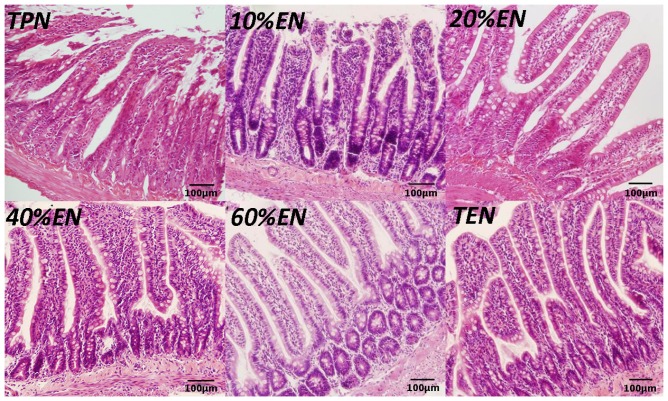
Representative sections of hematoxylin-and-eosin (H&E) staining of the proximal jejunum (×200). There are visible damage (e.g., subepithelial lifting, sloughing, and mucosal edema) in the villi of TPN and 10%EN groups. In the 20% and more EN groups, the rats’ intestinal epithelium had relatively normal histomorphology.

We found that a dosage of 20%EN (40 kcal/kg/day) had a visibly improvement of TJ protein claudin-1 expression (Figure 2A,B). Furthermore, we found that occludin and ZO-1, another two TJs of intestinal epithelial cells were significantly up-regulated in 40%EN and more EN groups (Figure 2C–F). Likewise, 20%EN protects the intestinal morphology. The results showed that TPN and 10%EN resulted in damage of normal TJ morphology and epithelial cell microvilli. On the contrary, in 20% and more EN groups, the structure of TJs and microvilli were relatively intact (Figure 3). Histological assessment of the small intestine samples revealed that there was severe damage in TPN and 10%EN groups (Figure 4).

#### 3.1.3. The 40%EN Improves Intestinal Goblet Cell Function

**Figure 5:**
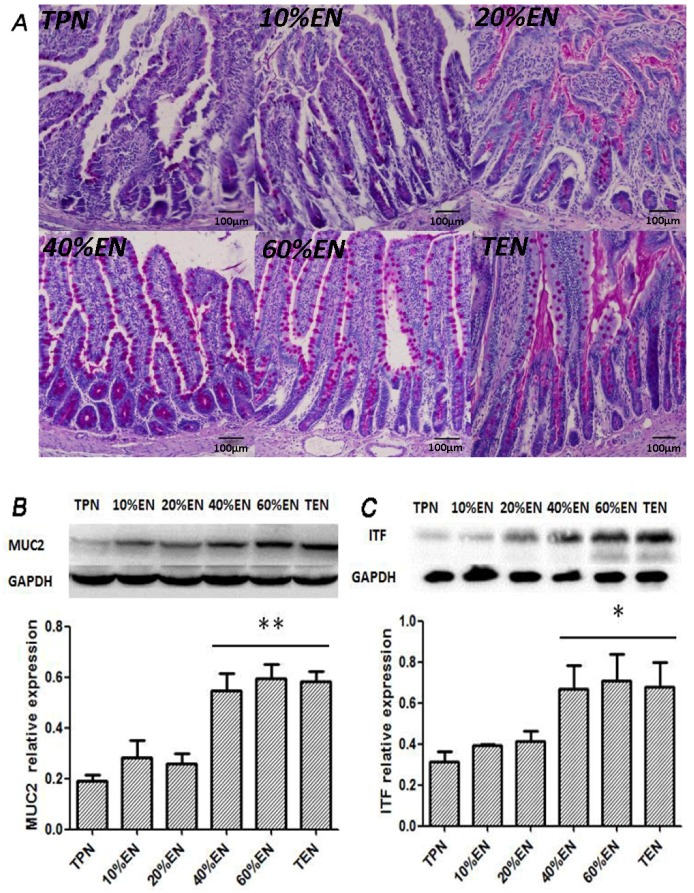
Goblet cell function analysis of in the distal ileum epithelium. (**A**) Periodic acid schiff (PAS) staining (purple red) of goblet cells (×200). The counts of goblet cells had no significant difference between groups (all the *p* < 0.05). However, there was an observable mucus secretion activity of cells in 40% and more EN groups; (**B**) Western blotting analysis of MUC2; (**C**) Western blotting analysis of ITF. MUC2 was significantly increased in 40%, 60%, and TEN groups compared with TPN (*p* = 0.006, 0.007, and 0.007, respectively). Parallel expression of ITF was also found among 40%, 60%, and TEN groups (*p* = 0.033, 0.042, and 0.029 compared with TPN). * *p* < 0.05, compared with TPN group; ** *p* < 0.01, compared with TPN group.

Although there is no difference in goblet cell counting (performed by two individual authors) in random 10 field of microscope of each group, we found that compared with TPN, 10%EN and 20%EN groups, 40%EN could increase the expression of MUC-2 and ITF (Figure 5B,C), which are the most two abundant protein/polypeptides expressed in goblet cells.

#### 3.1.4. Effects of 20%EN on Intestinal Epithelial Permeability in Intestinal IRI

**Figure 6:**
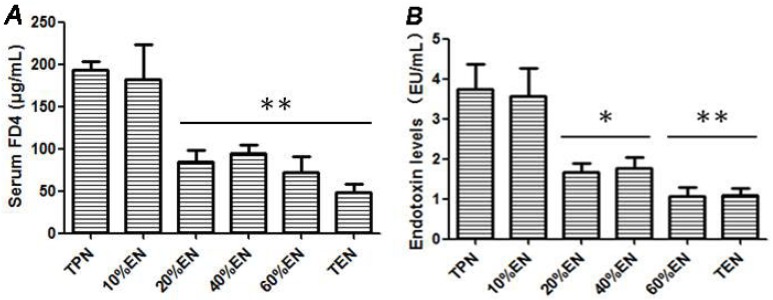
The analysis of intestinal barrier permeability. (**A**) Serum FD-4 level assay of in vivo test of epithelium permeability of rats. TPN and 10%EN groups had increased serum FD-4 concentrations. When EN dose was up to 20%, serum FD-4 was apparently decreased (*p* = 0.006), and it seems that this effect became relatively stable between 20% and 100%EN; (**B**) Mesenteric lymph nodes LPS assay of rats. A similar condition was found in LPS quantitative determination. It demonstrated that 20%EN is the minimally effective dose for maintaining the functional integrity of the intestinal barrier, which was mainly manifest in respect of the permeability of the macromolecule. * *p* < 0.05, compared with TPN group; ** *p* < 0.01, compared with TPN group.

In vivo intestinal permeability assay demonstrated that concentration of serum FD-4 was evidently lower in 20% and more EN groups (Figure 6A). This reflected that 20%EN had an improved effect in maintaining the intestinal barrier function. The endotoxin analysis also showed that the 20%EN group had a remarkably reduced endotoxin translocation in the mesenteric lymph node (Figure 6B). Normally, endotoxin was confined in the lumen by integrated epithelial tissue. When the barrier function was impaired, endotoxin could translocate via epithelium to other organs, commonly, mesenteric lymph nodes. It also demonstrated that 20%EN may have a potential advantage in other organs by preventing translocation of intraluminal content under intestinal IRI conditions.

### 3.2. Experiment 2

#### 3.2.1. Comparison of TPN, TPN+PDTC, TPN+YC-1, and 20%EN on Regulation of NF-κB/HIF-1α

**Figure 7:**
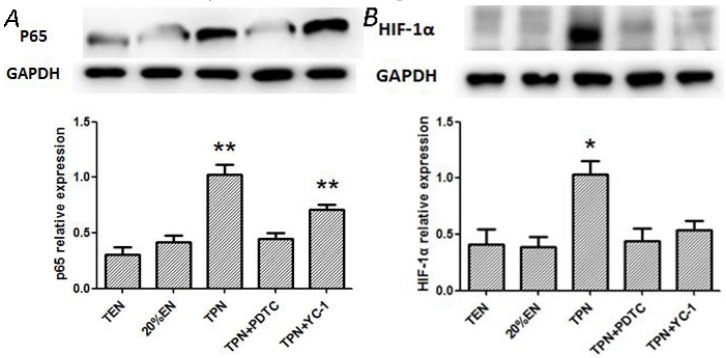
(**A**) Western blotting analysis of NF-κB-P65 in the intestine mucosa; (**B**) Western blotting analysis of HIF-1α in the intestine mucosa. We found a distinct upregulation of p65 in both TPN and TPN+YC-1 groups (*p* = 0.004 and 0.008 compared with TEN group). For HIF-1α expression, a significant increase was only found in the TPN group (*p* = 0.029 compared with TEN). * *p* < 0.05, compared with TEN group. ** *p* < 0.01, compared with TEN group.

Expression of P65 was significantly increased in TPN and TPN+YC-1 groups (Figure 7A) compared with other three groups (*p* < 0.01). YC-1 as a specific inhibitor of HIF-1α, which is a downstream signal molecule of P65, had a weak impact of the P65 protein activity. While expression of HIF-1α only increased in the TPN group compared with the other groups (Figure 7B). This may be because of the inhibition of P65, which results in the activation of HIF-1α which was decreased consequently. Although no statistical difference was found between 20%EN and TPN plus both inhibitors, a decreased tendency of P65 and HIF-1α expression was observed in 20%EN.

#### 3.2.2. Effects of 20%EN on Intestinal Epithelial Cell Claudin-1 Expression and Barrier Permeability

**Figure 8:**
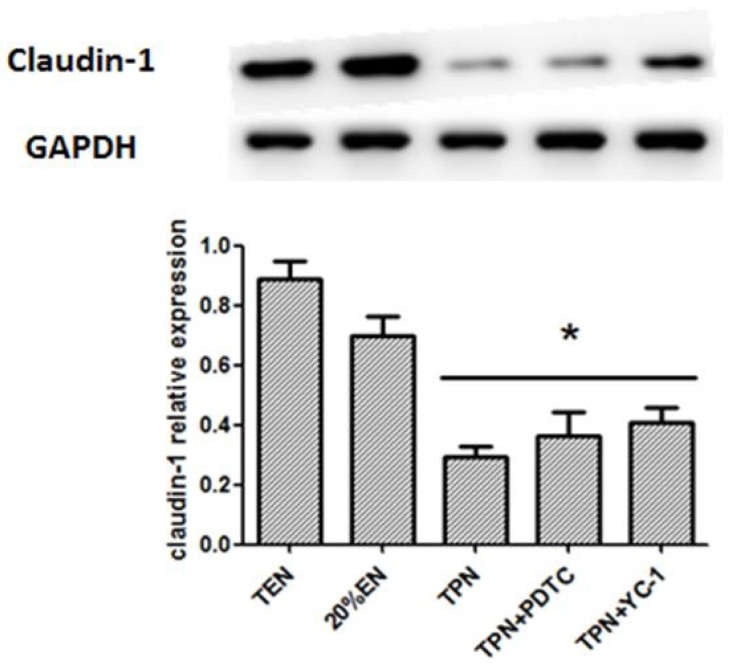
Western blotting analysis of claudin-1 in the intestine mucosa of all groups. It demonstrated obviously decreased claudin-1 in TPN, TPN+PDTC and TPN+YC-1 compared with TEN group (*p* = 0.022, 0.035 and 0.041, respectively). No statistical difference was found between the TEN and 20%EN groups. * *p* < 0.05, compared with TEN group.

**Figure 9:**
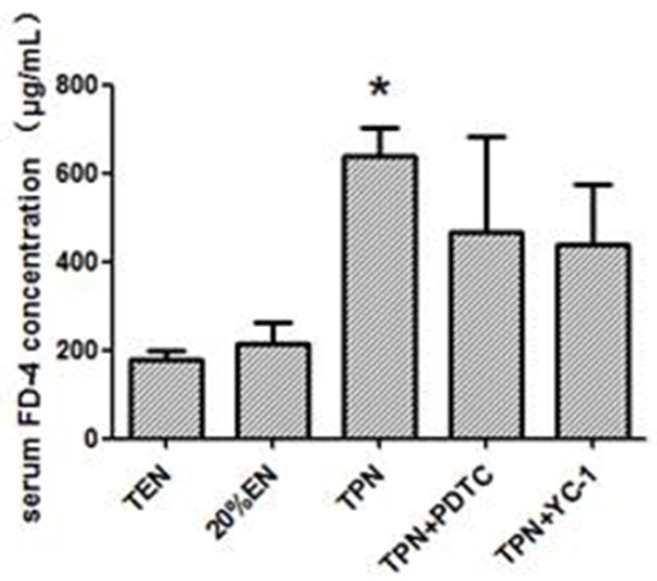
Serum FD-4 level assay of in vivo test of epithelium permeability among groups. Compared with TEN, only the TPN group had statistically higher epithelium permeability for the FD-4 molecule (*p* = 0.039). There were increasing trends of epithelium permeability in both TPN+PDTC and TPN+YC-1 groups compared with TEN, but no statistical significance was found. * *p* < 0.05, compared with TEN group.

Based on experiment 1, Claudin-1 had a significant up-regulation when the EN up to 20% of the full dose. The Western blot test demonstrated that Claudin-1 was apparently increased in TEN and 20%EN groups when compared with TPN, TPN+PDTC and TPN+YC-1 groups (Figure 8). We found that Claudin-1 was increased in 20%EN rats compared with TPN in experiment 1. However, when TPN combined with specific inhibitors of p65 and HIF-1α, the decreased Claudin-1 expression seemed still unchanged. In vivo intestinal permeability assay demonstrated that concentration of serum FD-4 was significantly enhanced only in the TPN group (Figure 9). This showed that 20%EN may exert a protective effect of the barrier function of the intestine.

#### 3.2.3. Effects of 20%EN on Preventing Cell Apoptosis, and Regulating Inflammatory Cytokines

**Figure 10:**
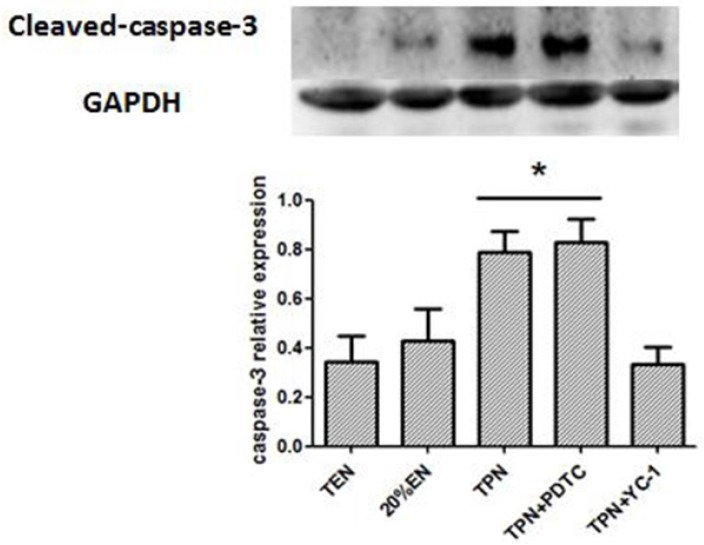
Western blotting analysis of cleaved caspase-3 in the intestine mucosa. Compared with TEN group, TPN and TPN+PDTC had increased cleaved caspase-3 expressions (*p* = 0.037 and 0.045). There is no difference among other groups. * *p* < 0.05, compared with TEN group.

**Figure 11:**
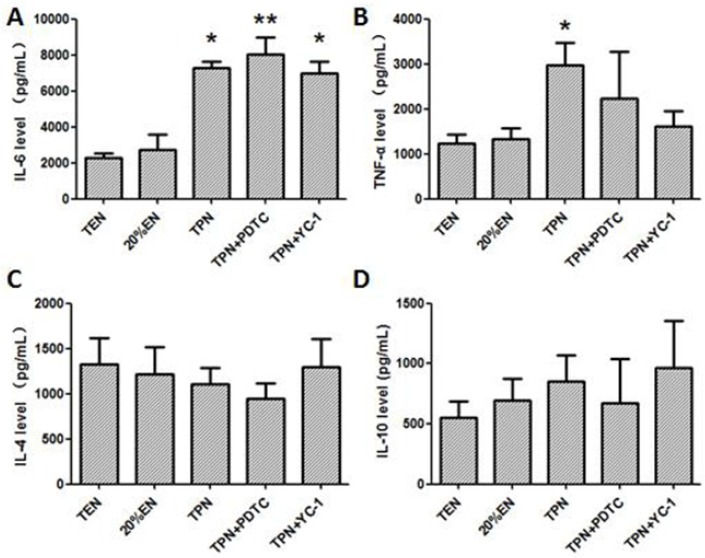
The analysis of inflammatory cytokine levels in intestinal epithelium tissue. (**A**) Enzyme-linked immunosorbent assay of TNF-α; (**B**) Enzyme-linked immunosorbent assay of IL-6; (**C**) Enzyme-linked immunosorbent assay of IL-4; (**D**) Enzyme-linked immunosorbent assay of IL-10. Both TNF-α and IL-6 were significantly upregulated in the TPN group compared with TEN (*p* = 0.023 and 0.030). IL-4 and IL-10 concentrations, however, showed no difference in all the groups. Additionally, TNF-α was increased in TPN+PDTC and TPN+YC-1 groups (*p* = 0.009 and 0.038 compared with TEN). * *p* < 0.05, compared with TPN group; ** *p* < 0.01, compared with TPN group.

The Western blot test showed that TPN and TPN+PDTC groups had an increased cleaved-caspase-3 expression (Figure 10), this may reflect an apoptosis-promoting potential cause by HIF-1α and p65 activation. Furthermore, it showed that blockade of HIF-1α may exert more effective anti-apoptotic activity compared with inhibition of NF-κB. Primary pro-inflammatory cytokines, TNF-α was visibly reduced in 20%EN and TEN groups (Figure 11A), and IL-6 was significantly increased in the TPN group alone (Figure 11B). These results demonstrated that TPN would lead to an inflammatory condition of intestinal tissue, which is harmful for the organs and may cause further damage of the intestinal barrier. However, we found no difference of IL-4 and IL-10 levels between groups (Figure 11C,D). These results suggested that 20%EN might play a better anti-inflammatory role for intestinal epithelium than specific inhibitors of NF-κB/HIF-1α.

## 4. Discussion

Intestinal epithelium injury and loss of natural barrier function are key elements in gut-derived systemic inflammatory response syndrome. The gut is a major target of injury during stress states and is a source of factors responsible for the development of remote organ dysfunction after major trauma and surgery [30]. In this study, we used a rat model of IRI to simulate major surgery patients. Different proportions of EN+PN were administered to the rats to determine the optimal dose for protecting the barrier function of the intestinal epithelium. We found that 20%EN had a reversal effect, preserving intestinal barrier function after IRI. Furthermore, our study showed that 40%EN improved goblet cell function and TJ protein expression. We also found that 20%EN might use an anti-apoptotic and anti-inflammatory mechanism. Downregulation of the NF-κB/HIF-1α pathway was observed and showed a similar trend to the changes in TJ expression and barrier permeability. Since previous studies have indicated that persistent activation of NF-κB/HIF-1α pathway leads to dysfunction of intestinal epithelial barrier in mice models [14,16] further researches should be performed, to investigate whether overactive NF-κB/HIF-1α pathway can be downregulated by enteral nutrients.

A number of factors have been confirmed to be associated with gut injury and inflammation, development of systemic inflammation, and distant organ dysfunction [31,32,33]. Apart from translocation of bacteria and endotoxins, these factors include cytokines and chemokines [34]. Studies investigating the mechanisms involved in the induction of these gut-derived inflammatory factors have primarily focused on the reperfusion inflammatory phase of gut injury. HIF-1 appears to be an early reperfusion-independent component in inflammatory response, and HIF-1 has been shown to be increased after an episode of hemorrhagic shock [35]. The induction of HIF-1α mRNA by NF-κB under normoxic conditions may be important because this process mainly results from dysfunction of the intestinal barrier and translocation of bacteria and LPS [36]. In our study, the TPN and 10%EN groups showed much higher NF-κB/HIF-1 expression even following normal blood perfusion after a 30-min period of acute mesenteric ischemia. Treatment with 20%EN was distinctly favorable for the intestinal barrier compared to the TPN and 10%EN groups. This finding demonstrates that 20% is the minimal effective dose of EN. This implies that IRI is an early activator of NF-κB/HIF-1α, and follow-up starvation may play an important role in NF-κB/HIF-1 expression, confirming the gut-injurious effect in intestinal IRI.

The NF-κB/HIF-1α signaling pathway is a crucial regulator of intestinal homeostasis and appears to have a dichotomous role in gut inflammatory disease [35,36]. A previous study showed that HIF-1 plays a maladaptive role in acute gut inflammatory and injury response in a 45-min intestinal I/R model, in contrast to reports that HIF-1 attenuates intestinal inflammation and induces the expression of gut barrier-protective genes in several chronic colonic inflammatory bowel disease models [37]. EN intolerance has been a challenge for doctors and nutritionists because the gut shows poor motility during disease. Thus, low-dose EN combined with SPN is a relatively optimum nutritional treatment for critically ill patients. In experiment 2, compared with the TPN and TPN+YC-1 groups, other groups exhibited significantly lower P65 expressions, while HIF-1α was only increased in the TPN group. A plausible explanation of these outcomes is that HIF-1α is one of the downstream molecules of NF-κB. Hence, blockade of NF-κB may decrease expression of p65, while HIF-1α can still be activated through other mechanisms such as cytokines or reactive oxygen species. It also implied that 20%EN, as well as TEN, would improve the prolonged expression of NF-κB/HIF-1α, which might consequently protect the barrier function of intestinal epithelium. Our further findings had suggested that 20%EN downregulated the apoptosis protein cleaved-caspase-3 and ameliorated the pro-inflammatory cytokines TNF-α and IL-6 in the intestinal epithelium, which are important products or mediators of the NF-κB/HIF-1α signaling pathway [38]. Since the normal equilibrium of cells proliferation/apoptosis is the crucial factor of intestinal epithelium barrier function, we assumed that 20%EN provided the essential calories to make the intestinal cells fulfill these physiological processes. This may be, at least in part, the mechanism of how 20%EN maintains barrier function in the gut.

The mucus layer is the first anatomical site at which the host encounters the gut microbiota. This layer creates a physical and chemical barrier to prevent the adherence of microbiota to the epithelium [39]. The properties of the mucus are derived from major gel-forming glycoprotein components known as mucins, which are continuously produced by specialized goblet cells found in large numbers in the epithelium of the entire gastrointestinal tract. MUC2 is the most abundantly expressed secretory mucin in the colon and is stored in bulky apical granules of the goblet cells, which form the characteristic goblet cell thecae [39]. Loss of the mucus layer in MUC2-deficient mice has been shown to increase the susceptibility to lethal infection induced by pathogenic and commensal flora [40,41]. ITF-peptide is the second-most abundant substance secreted by goblet cells after MUC2. Previous studies suggested that ITF plays an important role in the protection, repair, and healing of the gastrointestinal mucosa. Mice over-expressing ITF in the intestine exhibited increased resistance to intestinal damage and ulceration [42]. In contrast, mice with deleted ITF genes showed a higher susceptibility to gastrointestinal injury [42]. Grootjans et al. [39] reported that colonic ischemia disrupts the mucus layer facilitating bacterial penetration. This is rapidly counteracted by increased secretory activity of goblet cells, leading to the expulsion of bacteria from the crypts as well as restoration of the mucus barrier. However, a prolonged enteral dystrophy affects the goblet cells as well because of insufficient substrate and chronic inflammatory response. In this study, we found that compared to lower doses of EN (0%, 10%, and 20%), 40%EN may have a pro-synthetic effect in goblet cells after intestinal IRI despite the number of undifferentiated cells among all groups. Theoretically, goblet cells exhibit strong synthetic functions, increasing the need for nutritional substrates to exert these normal actions. Compared with columnar epithelium cells, goblet cells may require a relatively larger number of calories derived from intraluminal nutrients.

During the reperfusion phase, immune cells are in contact with bacteria or bacterial cell wall components such as LPS, causing NF-κB/HIF-1α pathway activation and HIF-1 accumulation. LPS activates p42/44 MAPK and subsequently NF-κB, inducing HIF-1α mRNA expression. Moreover, LPS-induced reactive oxygen species generation promotes HIF-1α protein accumulation [11]. Previous studies showed a deficiency in enteral nutrients complicating the intestinal epithelium under chronic inflammation, resulting in epithelial cell atrophy, TJ degradation, and immune cell dysfunction [21,22]. Cytokines are crucial mediators released during inflammation. For example, TNF-α activates HIF-1 via multiple pathways, including reactive oxygen species and nitric oxide production or NF-κB activation [33]. This may explain the TPN-induced NF-κB/HIF-1α persistent expression after intestinal IRI [20]. This complex network involves a number of factors such as hypoxia, inflammation, and immune response. Theoretically, EN has an improved effect of intestinal barrier function under physiological conditions, including after IRI. It was also confirmed in our study. However, the integrated mechanism of intestinal barrier function is complex, involving massive inflammatory and immunologic factors. Although we have some novel findings which imply that the EN-related protective effect has correlation with NF-κB/HIF-1α pathway at some level, this hypothesis is still unclear due to absence of direct evidence. Apart from the subtle barrier mechanism and elusive molecular network during inflammation, we thought the physiopathologic discrepancies between human and animals are one of the most important factors that impact the results. Hence the primary limitation of our study is that we used the TEN group rats as positive control. This could hardly be performed in patients because a critically ill patient could scarcely tolerate the full dose of EN under normally clinical conditions. The result must be interpreted more cautiously, and further tests should be performed.

## 5. Conclusions

In conclusion, our study showed that intestine IRI led to a dysfunction of the intestinal barrier, which exposed the intestinal epithelium directly to the intraluminal content. It demonstrated that 20%EN exerted an improved effect of intestinal barrier function. In addition, 40%EN improved goblet cell function and TJs expression, which are other important components of the intestinal epithelium barrier.
